# Establishing a Predictive Nomogram for Cervical Lymph Node Metastasis in Patients With Papillary Thyroid Carcinoma

**DOI:** 10.3389/fonc.2021.766650

**Published:** 2022-01-19

**Authors:** Qiao Hu, Wang-Jian Zhang, Li Liang, Ling-Ling Li, Wu Yin, Quan-Li Su, Fei-Fei Lin

**Affiliations:** ^1^ Department of Ultrasound, The People’s Hospital of Guangxi Zhuang Autonomous Region & Guangxi Academy of Medical Sciences, Nanning, China; ^2^ School of Public Health, Sun Yet-Sen University, Guangzhou, China; ^3^ Department of Pathology, The People’s Hospital of Guangxi Zhuang Autonomous Region & Guangxi Academy of Medical Sciences, Nanning, China

**Keywords:** nomogram, papillary thyroid carcinoma, cervical lymph node, metastasis, predictor

## Abstract

**Objectives:**

The purpose of this study was to establish a nomogram for predicting cervical lymph node metastasis (CLNM) in patients with papillary thyroid carcinoma (PTC).

**Materials and Methods:**

A total of 418 patients with papillary thyroid carcinoma undergoing total thyroidectomy with cervical lymph node dissection were enrolled in the retrospective study from January 2016 to September 2019. Univariate and multivariate Logistic regression analysis were performed to screen the clinicopathologic, laboratory and ultrasound (US) parameters influencing cervical lymph nodes metastasis and develop the predicting model.

**Results:**

CLNM was proved in 34.4% (144/418) of patients. In the multivariate regression analysis, Male, Age < 45 years, Tumor size > 20mm, multifocality, ambiguous boundary, extracapsular invasion and US-suggested lymph nodes metastasis were independent risk factors of CLNM (*p* < 0.05). Prediction nomogram showed an excellent discriminative ability, with a C-index of 0.940 (95% confidence interval [CI], 0.888-0.991), and a good calibration.

**Conclusion:**

The established nomogram showed a good prediction of CLNM in patients with PTC. It is conveniently used and should be considered in the determination of surgical procedures.

## Introduction

Papillary thyroid carcinoma (PTC) originates from the thyroid follicular epithelium and is the major pathological type among thyroid malignancies, accounting for approximately 80% of all thyroid cancers (1). PTC is more common in female patients, and the incidence rate in women is three times that in men. Although PTC is considered to be an indolent tumor with low malignancy, slow disease progression, and good prognosis (2), 20–50% of patients still have early cervical lymph node metastasis (CLNM), leading to a high risk of local recurrence (3).

CLNM of PTC usually manifests as sequential lymph node metastasis from the central area to the lateral cervical area (4). However, some PTCs may undergo direct lateral lymph node metastasis (LLNM) without central lymph node metastasis, which is called “skip metastasis” (5). CLNM is the strongest risk factor for local recurrence and the prognosis of PTC patients and is an important indicator for determining the surgical approach before surgery (6).

Prophylactic cervical lymph node dissection (CLND) has always been controversial (7, 8). Some scholars believe that CLND can change the tumor–node–metastasis (TNM) staging of some patients, and reduce their risk of postoperative recurrence (9). Other studies suggest that there is still not enough evidence to show that prophylactic CLND is beneficial in reducing the recurrence rate and improving the prognosis, and it increases the risk of potential surgical complications such as recurrent laryngeal nerve injury and reduced parathyroid function (10). Therefore, it is necessary to efficiently and accurately assess the presence or absence of CLNM before surgery.

Ultrasound has the advantages of real-time, noninvasive, dynamic, and simple operation and has become the preferred tool for cervical lymph nodes examination. However, due to the complex anatomical location of cervical lymph nodes and interference by air echoes in the trachea, the detection rate of central lymph node metastasis by ultrasound is very limited, and the sensitivity of conventional ultrasound in the diagnosis of central lymph node metastasis is low, at only 20–33% (11). In addition, ultrasound cannot detect some occult lymph nodes metastasis. In recent years, some scholars have attempted to use clinical data and imaging data to assess the risk faced by PTC patients before surgery and screen out the patients most likely to develop CLNM to compensate for the low sensitivity of conventional ultrasound at directly diagnosing CLNM. Hu et al. (5) used clinicopathological data to analyze the risk factors for skip metastasis in PTC patients. Their results suggest that age > 55 years, tumor located in the upper portion, and unilaterality were independent risk factors of skip metastasis. In another study, a radiomic model established based on anatomical and functional magnetic resonance images was used to screen independent risk factors for CLNM in PTC patients (12). Tong et al. (13) established a nomogram model based on the central lymph node status suggested by conventional ultrasound and computed tomography (CT) images, which could be used to predict LLNM in PTC patients before surgery. No previous reports have established a quantitative risk assessment model for the screened risk factors, and there is no unified method for the preoperative prediction of CLNM in PTC patients. Thus, the purpose of our study was to establish a nomogram model for the preoperative prediction of CLNM of PTC based on clinical, pathological, and ultrasound imaging characteristics and to test its predictive efficacy.

## Materials and Methods

### Patients

This study was performed with the approval of the Ethics Committee of the People’s Hospital of Guangxi Zhuang Autonomous Region, China (IRB No. KY-KJT-2019-04). The informed consent requirement was obtained from all participants. A total of 418 PTC patients (109 males and 309 females, aged 9-75 y with a median age of 43 y) were enrolled between January 2016 and September 2019. Inclusion criteria: ① preoperative thyroid fine-needle aspiration biopsy or postoperative pathology diagnosed PTC; ② complete clinical, pathological, and conventional ultrasound image data; ③ first thyroid surgery (thyroid lobectomy or any type of thyroidectomy) and undergoing CLND (at least central lymph nodes dissection); and ③ pathology confirming the presence or absence of lymph node metastasis. The exclusion criteria were as follows: ① non-PTC pathology; ② other treatments before surgery (such as iodine-131 or surgical history); ③ incomplete ultrasound, clinical, or pathological data; ④ distant metastasis; and ⑤ malignant tumors at other sites. The clinical, serological, and pathological data of the included cases were retrospectively analyzed, including age (<45 years, 45-55 years, or >55 years), sex, bilaterality (unilateral or bilateral), tumor size (maximum diameter <10 mm, 10-20 mm, or >20 mm), Hashimoto’s thyroiditis (absent or present), serum thyroid-stimulating hormone (TSH), thyroid peroxidase antibodies (TPOAb), triiodothyronine (T3), thyroxine (T4), free triiodothyronine (FT3), free thyroxine (FT4), and the expression of galectin-3, cytokeratin (CK)-19, and CK-34 in tumor specimens.

The surgical range of cervical lymph node dissection of the enrolled patients were determined based on the preoperative fine-needle aspiration cytological examination and/or the intraoperative rapid frozen pathological results: ① unilateral PTC: excision of the affected lobe plus isthmus and lymph nodes dissection of the ipsilateral central region (level VI);  x2461; bilateral lymph nodes dissection in the central region for patients with isthmus or bilateral PTC; ③ lymph nodes dissection of the affected lateral cervical region if preoperative ultrasound reported lymph node metastasis in the lateral cervical region (levels II, III, and IV) and fine-needle aspiration cytology indicated suspicious positivity; ④ additional lymph node dissection in level V if lymph node metastasis in level V was suspected.

### Ultrasonography Imaging

**Figure 1:**
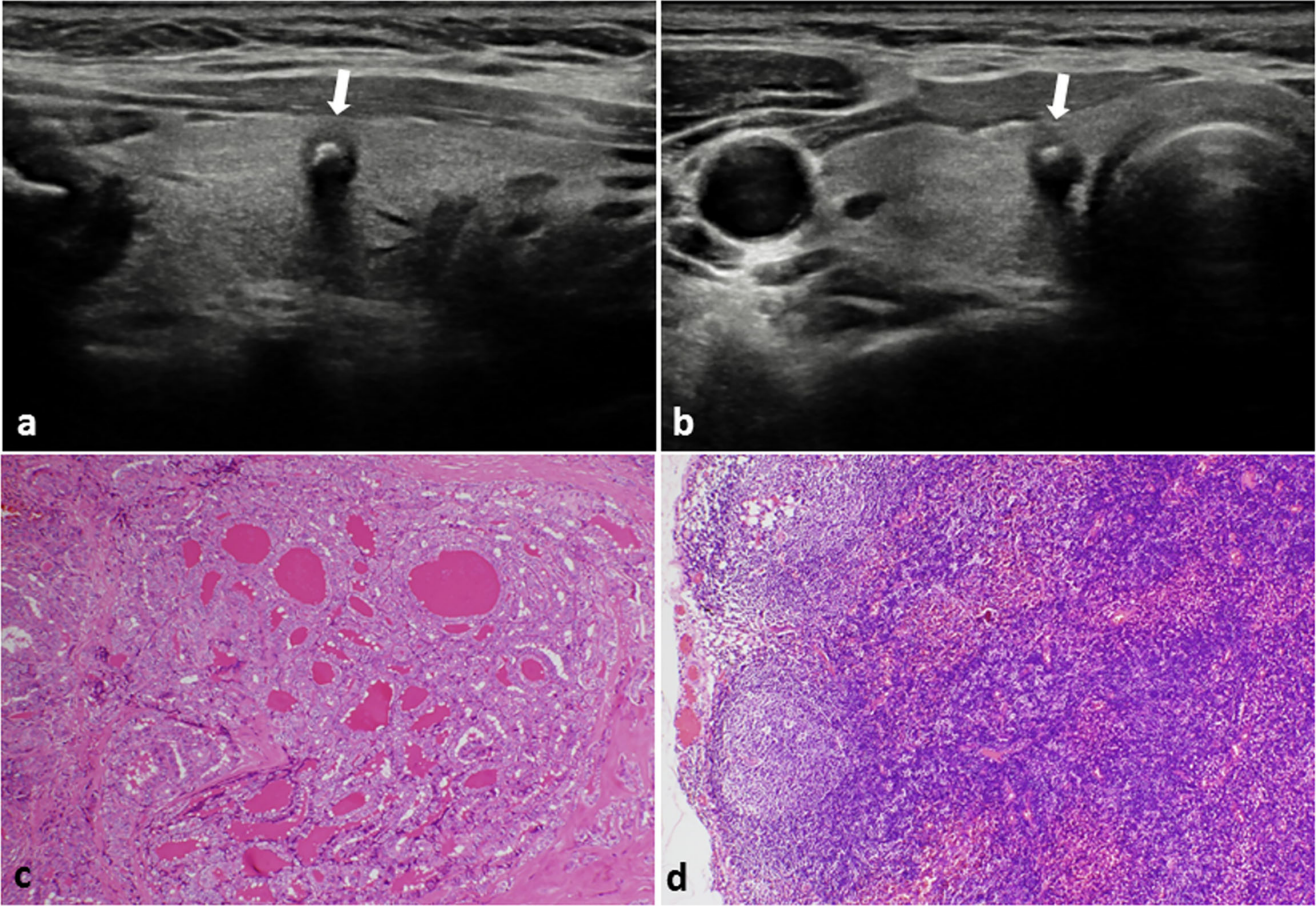
PTC with negative CLNM in a 48-year-old female. **(A)** Longitudinal sonogram and **(B)** Transverse sonogram showing a 5×5×6 mm hypoechoic nodule in the right lobe of thyroid (arrow). The nodule was regular, legible, with a taller than wide shape and absence of extracapsular invasion. A bulky calcification (> 1 mm) was observed inside the nodule. Histological examination of the tumor **(C)** and lymph nodes **(D)** indicated no lymph nodes metastasis in the central area of the neck. H-E × 100.

**Figure 2:**
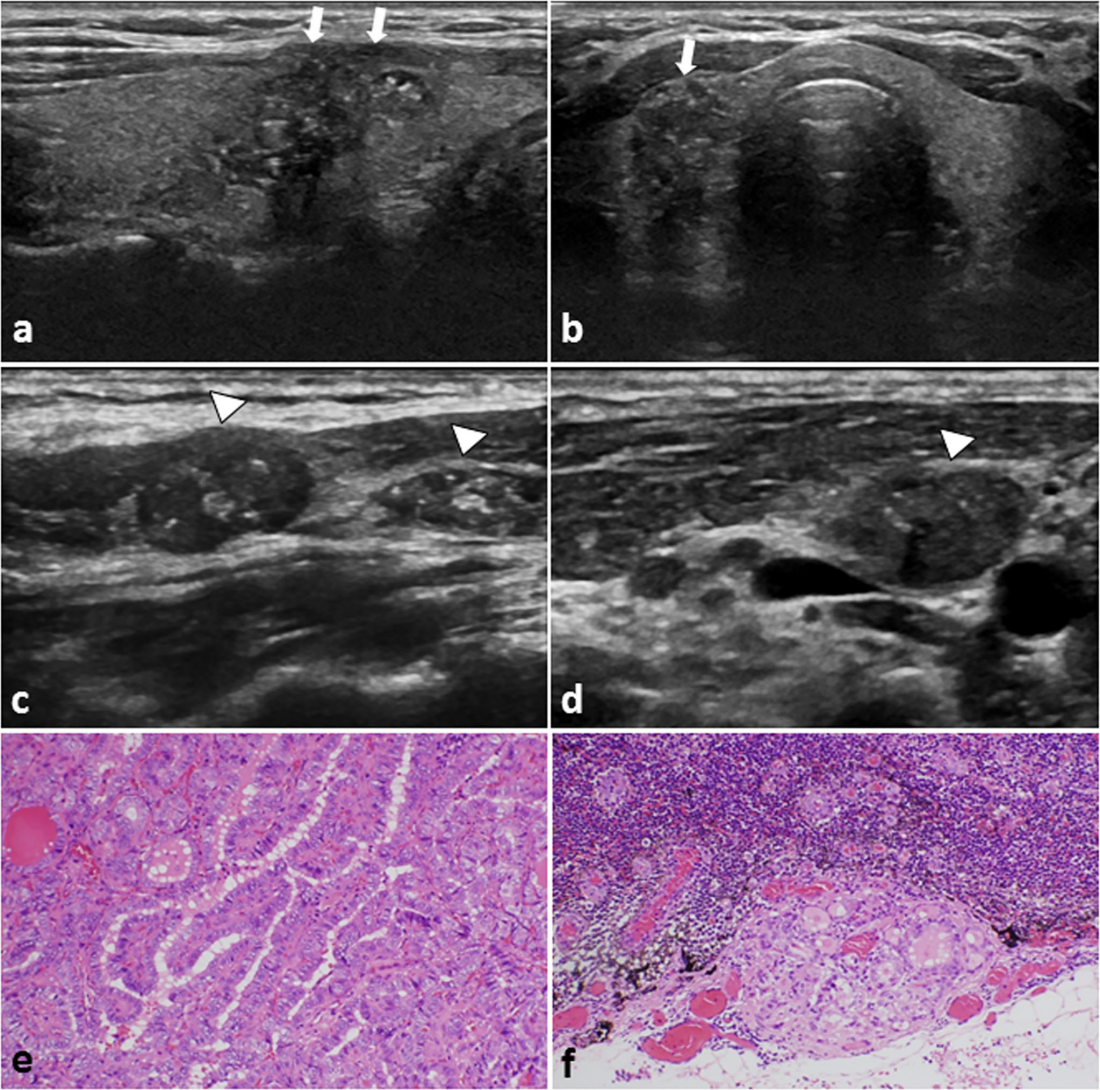
A 29-year-old female PTC patient with positive CLNM. **(A)** Longitudinal sonogram and **(B)** Transverse sonogram showing a hypoechoic lesion in the right lobe of thyroid (arrows). The tumor size is 26×17×16mm, irregular, ambiguous, had multiple microcalcification (≤ 1 mm) inside, and hard to be distinguish from the anterior thyroid capsule. Conventional US of the lymph nodes on **(C)** Level III and **(D)** level IV area of the neck. The lymph nodes were approximately rounded, lymphatic hilus structure disappeared, and with scattered calcification inside (arrowheads). These features on US suggest the cervical lymph nodes metastasis. Histological examination confirmed the **(E)** thyroid papillary carcinoma in the right lobe, and **(F)** revealed lateral cervical lymph nodes metastasis. H-E × 200.

Preoperative conventional ultrasound examination was performed using a GE Logiq E9 ultrasound system (GE Healthcare Life Sciences, Chicago, IL, USA) with a 6-15 MHz linear transducer. The patients were placed in the supine position with the neck extended. The thyroid and cervical lymph nodes were scanned on multiple sections, and their characteristics were recorded, including tumor position (left, right, others), internal component (solid, not solid), echogenicity (hypoecho, not hypoecho), taller than wide (absent or present), multifocality (single or multiple), margin (regular or irregular), boundary (legible or ambiguous). The presence or absence of microcalcification (defined as a maximum diameter of calcification ≤1 mm) and the presence or absence of extracapsular invasion (defined as contact between the nodule and the anterior and/or posterior capsule of the thyroid, such that the continuity of the capsule line was interrupted or obscured by nodules that could not be explored), and the sonographic assessment of the cervical lymph nodes (**Figures 1**, **2**). Blinded to the clinical and pathological information, two ultrasound physicians with more than 2 years of experience were responsible for the interpretation of sonographic images. In cases of discrepancies, the two physicians reanalyzed and discussed together to reach a consensus.

### Statistical Analysis

R language 3.5.3 statistical software was used for data analysis. Continuous variables were presented as mean ± standard deviation (SD), and comparisons between groups were performed with Student’s *t-* test or analysis of variance. Categorical data were expressed as the number of cases or percentages (%), and comparisons between the groups were performed with the Chi-square test. The risk factors with *P* < 0.1 were screened by univariate analysis and were included in the multivariate logistic regression analysis. The stepwise regression was used to identify the independent predicting factors for CLNM in PTC patients, from which a nomogram model was plotted and established (14). The concordance index (C-index) and 95% confidence interval (CI) were used to evaluate the discriminability of the nomogram model. The range of the C-index is 0–1. The closer the C-index is to 1, the better the model differentiates patients and the more accurate the predictive performance. The calibration curve was used to evaluate the agreement between the predicted probability of CLNM in the nomogram of PTC and the actual probability value. The X-axis represents the predicted probability calculated by the nomogram, and the Y-axis represents the actual pathological assessment of lymph node metastasis. Falling on the 45° diagonal reference line indicated that the prediction was in good agreement with pathology. *P*<0.05 indicated that a difference was statistically significant.

## Results

The demographic data of patients are shown in **Table 1**. Out of 418 patients, postoperative pathological results showed that 144 patients (144/418, 34.4%) had positive CLNM, of whom 99 cases had central lymph node metastasis, 28 cases had central and lateral lymph node metastasis, and 17 cases had skip metastasis of LLNM; 274 cases (274/418, 65.6%) were negative for CLNM.

**Table 1:** Patients characteristics.

	Case number	Central lymph node metastasis		Positive rate	*P-*Value
		Negative	Positive		
Gender					
Female	309	215	94	30.42%	0.006
Male	109	59	50	45.87%	
Age					
< 45 years	230	139 (50.73%)	91	39.57%	0.051
45-55 years	109	80 (29.20%)	29	26.61%	
> 55 years	79	55 (20.07%)	24	30.38%	
TPOAb	418	46.11 ± 137.83*	54.54 ± 169.68*		0.376
TSH	418	1.96 ± 1.98*	1.98 ± 1.59*		0.323
T3	418	1.47 ± 0.39*	1.48 ± 0.32*		0.303
T4	418	101.12 ± 19.40*	101.40 ± 20.98*		0.426
FT3	418	5.35 ± 0.97*	5.36 ± 0.91*		0.895
FT4	418	11.72 ± 2.73*	11.33 ± 2.28 *		0.725
Multifocality					
Multiple	115	55	60	52.17%	<0.001
Single	303	219	84	27.72%	
Galectin-3					
–	21	18	3	14.29%	0.047
+	397	257	140	35.26%	
CK19					
–	11	8	3	27.27%	0.729
+	407	265	142	34.89%	
CK34					
–	22	16	6	27.27%	0.493
+	396	258	138	34.85%	
Position					
Others	74	37	37	50.00%	0.008
Left lobe	153	112	41	26.80%	
Right lobe	191	125	66	34.55%	
Hashimoto’s thyroiditis					
Absent	327	216	111	33.94%	0.724
Present	91	58	33	36.26%	
US suggested CLNM					
Absent	290	253	37	12.76%	<0.001
Present	128	21	107	83.59%	
Tumor size					
<10mm	185	156	29	15.68%	<0.001
10-20mm	135	89	46	34.07%	
>20mm	98	29	69	70.41%	
Component					
Solid	378	245	133	35.19%	0.400
Solid cystic	40	29	11	27.50%	
Echogenicity					
Hypoecho	401	259	142	35.41%	0.072
Isoecho	17	15	2	11.76%	
Taller than wide					
Absent	302	185	117	38.74%	0.005
Present	116	89	27	23.28%	
Margin					
Irregular	177	93	84	47.46%	<0.001
Regular	241	181	60	24.90%	
Boundary					
Ambiguous	223	116	107	47.98%	<0.001
Legible	195	158	37	18.97%	
Microcalcification					
Absent	92	75	17	18.48%	<0.001
Present	326	199	127	38.96%	
Extracapsular invasion					
Absent	292	239	53	18.15%	<0.001
Present	126	35	91	72.22%	
bilaterality					
Absent	353	246	107	30.31%	<0.001
Present	65	28	37	56.92%	

*Mean ± standard deviation.

TPOAb, Thyroid Peroxidase antibody; TSH, thyroid stimulating hormone; T3, triiodothyronine; T4, thyroxine; FT3, free triiodothyronine; FT4, free thyroxine; US, ultrasound; CLNM, cervical lymph node metastasis.

### Univariate Analysis of Risk Factors for CLNM

Univariate analysis showed that when comparing the CLNM-positive and -negative groups, there were statistically significant differences in factors such as gender, age, expression of galectin-3, tumor size, boundary, margin, bilaterality, taller than wide, multifocality, tumor position, echogenicity, presence of microcalcification, extracapsular invasion, and US suggested CLNM (*P* < 0.1). There was no significant difference in the expression level of TSH, TPOAb, T3, T4, FT3, FT4, CK-19, and CK-34, tumor internal components, and the presence or absence of thyroiditis between the CLNM-positive group and the CLNM-negative group (*P* > 0.1) (**Table 2**).

**Table 2:** Univariate analysis of risk factors associated with CLNM in PTC patients.

Factors		OR	95% CI		*P-*Value
Gender	(male vs. female)	1.938	1.238	3.034	0.004
Age					
	<45 years	1			
	45-55 years	0.554	0.336	0.9136	0.021
	>55 years	0.667	0.386	1.153	0.146
TPOAb(U/mL)		0.782	0.508	1.206	0.266
TSH(uIU/mL)		1.005	0.898	1.125	0.926
T3 (nmol/L)		1.075	0.605	1.909	0.805
T4 (nmol/L)		1.000	0.990	1.011	0.896
FT3 (pmol/L)		1.0111	0.8121	1.257	0.925
FT4 (pmol/L)		0.938	0.860	1.024	0.155
Multifocality	(single vs. multiple)	2.844	1.824	4.434	<0.001
Galectin	(negative vs. positive)	4.354	0.985	19.247	0.052
CK19	(negative vs. positive)	1.619	0.322	8.146	0.559
CK34	(negative vs. positive)	1.492	0.529	4.239	0.453
bilaterality	(unilateral vs. bilateral)	3.0381	1.7689	5.218	<0.001
Position					
	left lobe	0.466	0.196	1.1087	0.084
	right lobe	0.672	0.289	1.563	0.356
	others	1.439	0.546	3.790	0.462
Hashimoto’s thyroiditis	(absent vs. present)	1.107	0.682	1.798	0.681
US suggested CLNM	(absent vs. present)	34.840	19.483	62.304	<0.001
Tumor size					
	<10mm	1			
	10-20mm	2.780	1.632	4.736	<0.001
	>20mm	13.256	7.337	23.952	<0.001
Component	(solid vs. solid cystic)	0.6987	0.338	1.443	0.333
Echogenicity	(hypoechoic vs. others)	0.243	0.0548	1.079	0.063
Taller than wide	(absent vs. present)	0.4797	0.2947	0.7827	0.003
Margin	(regular vs. irregular)	0.3677	0.2427	0.556	<0.001
Boundary	(ambiguous vs. legible)	3.939	2.527	6.141	<0.001
Microcalcification	(absent vs. present)	2.816	1.589	4.986	<0.001
Extracapsularinvasion	(absent vs. present)	11.724	7.179	19.146	<0.001

OR, odds ratio; PTC, papillary thyroid carcinoma; US, ultrasound; CLNM, cervical lymph node metastasis.

### Multivariate Analysis of Risk Factors for CLNM

The risk factors with statistically significant differences in the univariate analysis were included in the multivariate logistic regression analysis. Seven variables (including male sex, age <45 years, tumor maximum diameter > 20mm, multifocality, ambiguous boundary, extracapsular invasion, and US suggested CLNM) were proved to be independent predicting factors associated with CLNM (**Table 3**). The ranking of the odds ratio (OR) value was as follows: US suggested CLNM > tumor size > extracapsular invasion > gender > multifocality > boundary > age.

**Table 3:** Multivariate analysis of predictive factors associated with CLNM in PTC patients.

Variables	OR	95% CI	*P-*Value
Gender (Male)	2.823	1.269-6.279	0.011
Age (<45 years)	1.806	1.095-2.979	0.021
Multifocality	2.666	1.225-5.802	0.013
US suggested CLNM	33.192	14.867-74.101	<0.001
Tumor size (10-20mm)	1.833	0.795-4.223	0.155
Tumor size (>20mm)	6.635	2.541-17.324	<0.001
Boundary (ambiguous)	2.046	1.002-4.414	0.042
Extracapsular invasion	5.532	2.556-11.971	<0.001

OR, odds ratio; PTC, papillary thyroid carcinoma; US, ultrasound; CLNM, cervical lymph node metastasis.

### Establishment of Nomogram Model

**Figure 3:**
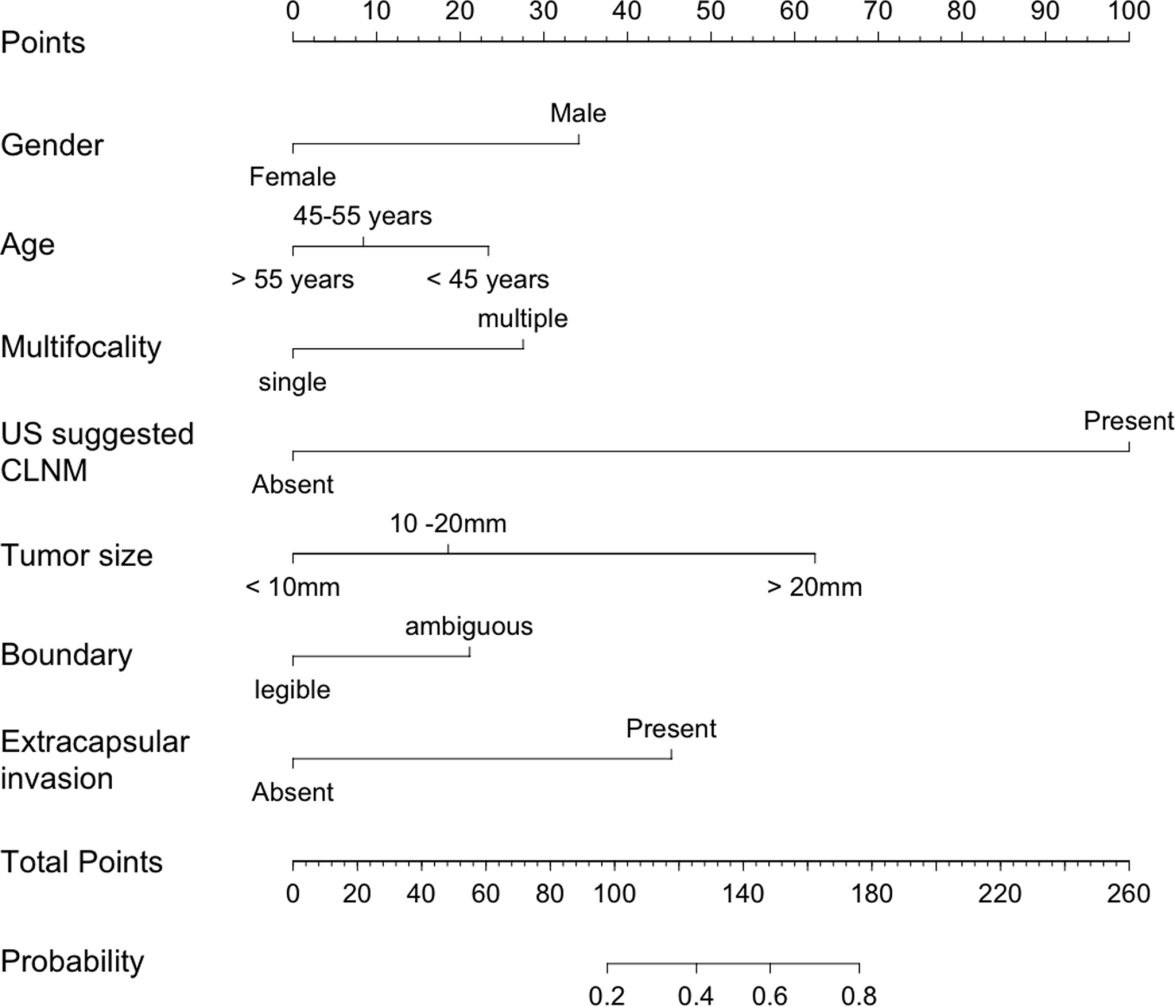
Nomogram for predicting CLNM in patients with papillary thyroid carcinoma. CLNM, cervical lymph node metastasis; US, ultrasound.

**Figure 4:**
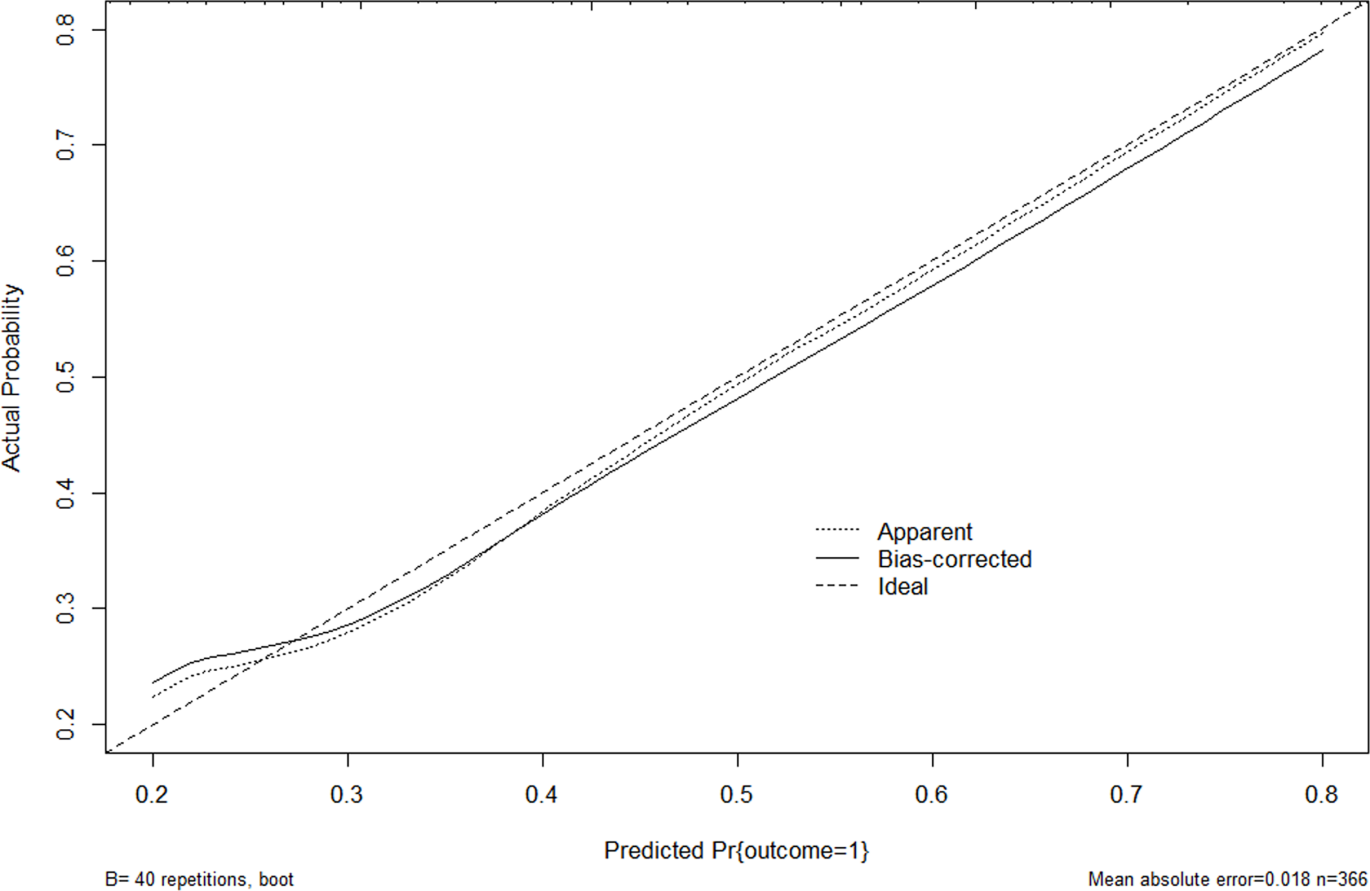
Calibration curve for internal validation of the CLNM nomograms. Nomogram predicted CLNM probabilities are plotted on the x-axis; actual CLNM probabilities are plotted on the y-axis. The dashed line along the 45-degree line passing through the point of origin represents a perfect calibration model in which the predicted probabilities are consistent with the actual probabilities. US, ultrasound; CLNM, cervical lymph node metastasis.

The nomogram model was established using the seven independent risk predictors: gender, age, multifocality, tumor size, extracapsular invasion, boundary, and US suggested CLNM (**Figure 3**). The model score axis (2-9) corresponded to the score of each predictor from bottom to top, and then the total score was calculated to find the risk of CLNM corresponding to the last risk axis. The C-index of the nomogram was 0.940 (95% CI, 0.888-0.991), suggesting that the nomogram model has a favorable prediction performance of CLNM. The calibration curve displayed good fitting with the 45° reference line suggesting that the predictive model was in good consistency with the actual condition of lymph node metastasis (**Figure 4**).

## Discussion

Nomogram model is a predictive tool, which uses a visually graphical representation of a statistical predictive model that to generate a numerical probability of a clinical event (15). Nomograms are widely used to predict clinical disease onset and patient prognosis, especially in the field of cancer (16). Tong et al. (13) proposed a radiomics nomogram, which incorporated the radiomics signature and the ultrasound and contrast enhanced CT-reported cervical lymph node status, for the prediction lateral lymph node metastasis in patients with PTC. The radiomic nomogram demonstrated a best predictive performance than either the radiomic signature or the US- or CT- reported lateral cervical lymph node status with an area under the ROC curve and a C-index of 0.914 (95% CI, 0.842-0.987) and 0.927 (95% CI, 0.856-0.980), respectively. However, CT has the limits of radiation and costly, and has a risk of contrast agent allergy. It has been reported that the incidence of hypersensitivity reactions related to iodinated contrast media ranges from 0.004–3.0% (17). Several scholars recommended CT as a complementary modality for detecting the extent of tumor involvement in the airway or esophagus as well as detecting extra nodal tumor extension (18). In our present study, a quantitative nomogram model for the preoperative prediction of CLNM of PTC was established based on laboratory serum indicators, pathology, and non-invasive ultrasound imaging characteristics. This nomogram model exhibited a promising value with an excellent discriminative ability of a C-index of 0.940 (95% CI, 0.888-0.991), and a good calibration.

The results of the present study showed that male sex, age <45 years, multifocality, tumor maximum diameter >20 mm, extracapsular invasion, ambiguous boundary, and lymph node metastasis suggested by ultrasound were independent risk predictors for CLNM of PTC. The risk of CLMN in male patients was 2.82 times that of female patients. Some scholars believe that men (especially young men) have a high basal metabolism, which accelerates the spread of tumors and puts them at greater risk for CLNM (19). This finding is consistent with the results of Gui et al. (20). PTC can occur in various age groups, with a high incidence in 30–60 years old. This study analyzed the relationship between age and CLNM and found that the risk of CLNM in PTC patients <45 years old was significantly higher than that in other age groups (*P*=0.021). This is consistent with the conclusion of Zheng et al. (21) that age is an independent risk factor for the development of CLNM of PTC. However, Xue et al. (22) reported no difference in lymph nodes metastasis between patients <45 years old and patients ≥45 years old. The explanation for the inconsistent results may be related to the relatively small sample size and the bias of case selection in the study of Xue et al.

Multiple lesions are a clinicopathological feature of PTC, having an incidence of approximately 23–40% in PTC patients. Compared with single lesions, multifocal PTC is more invasive, and the more cancer lesions a person has, the higher their degree of malignancy, the more prone they are to lymph node metastasis, and the poorer the prognosis will be (23). Cyclooxygenase-2 (COX-2) and vascular endothelial growth factor (VEGF) are highly expressed in multifocal PTC (24). High expression of COX-2 and VEGF is closely related to the extent of PTC invasion and lymph node metastasis. COX-2 can catalyze the synthesis of a large amount of prostaglandin E2 (PGE2), which not only promotes cell proliferation and inhibits apoptosis but also promotes tumor angiogenesis. VEGF promotes neovascularization, increases vascular permeability, and plays an important role in tumor nutrient transport before angiogenesis. The results of present study also showed that the risk of CLNM of multifocal PTC was 2.67 times that of a single lesion. A study on multifocal PTC found that multiple lesions could be accurately identified in 75.9% of cases by ultrasonography (25). There was no significant difference between their multifocal findings determined by ultrasound and their histopathological findings. Therefore, the prediction model based on ultrasound features is reliable and feasible.

Tumor size has always been considered an important predictor of CLNM in patients with PTC. The larger the tumor volume, the higher the risk of CLMN. The mechanism may be related to the overexpression of VEGF. With the enlargement of nodules, the intra-tumor vessels are induced by various angiogenic factors to proliferate rapidly, forming many disorganized vascular networks. The active angiogenesis of the tumor increased the risk of CLNM (26, 27). However, the prediction thresholds reported by previous studies are different. Ahn et al. (28) reported that a tumor diameter ≥ 1 cm was a risk factor for CLNM, while Yan et al. (29) believed that tumor diameter had predictive value when it was ≥ 0.25 cm. The results of this present study found that the risk of developing CLNM of tumors with a maximum diameter of 10–20 mm and >20 mm was 1.83 and 6.63 times that of tumors with a maximum diameter of < 10 mm.

Extracapsular invasion of the thyroid is another independent predictor for CLNM (30). Kamaya et al. (31) reported that the criteria for ultrasound assessment of capsule invasion in PTC were that the nodule was adjacent to the capsule (i.e., there was no normal thyroid tissue between the nodule and the capsule), regardless of whether the capsule was interrupted. Jin et al. (32) suggested that the risk of CLNM was higher when the contact area between the nodule and the capsule was greater than 25%. Wang et al. (33) showed that tumor invasion and breakthrough of the thyroid capsule increased the likelihood of invading the lymphatic ducts and increased the risk of CLNM, and the distance between the tumor and the capsule was negatively correlated with the rate of lymph node metastasis. In our present study, the risk of CLNM in PTC patients with extracapsular invasion was significantly higher than that of patients without extracapsular invasion, which is consistent with the results of previous studies

The results of this study also indicated that an ambiguous boundary is an important factor for predicting lymph node metastasis, which is in line with the report of Xue et al. (22). Tumor metastasis required cancer cells invasion into the stroma, migration into the vessels, and proliferation in the lymph nodes (34).The invasive growth characteristics of the tumor cause the loss of a clear boundary between the tumor and the surrounding normal tissues.

On ultrasound, metastatic lymph nodes of PTC is recognized as round-like, calcified, cystic, and disordered or absent lymphatic hilus structure. In this study, any of the above changes detected by ultrasound examination was suspected lymph node metastasis. Ultrasound suggested lymph node metastasis accounted for the highest score in the nomogram model established in the present study, which may become one of the most important indicators for the prediction of CLNM.

Microcalcification is the deposition of calcium salts caused by vascular and fibrous hyperplasia, which reflects the rapid growth of cancer cells and is a typical ultrasound manifestation of PTC. However, the effect of microcalcification on CLNM of PTC is controversial. The presence of microcalcifications, especially the presence of diffuse microcalcifications, has been highly correlated with CLNM, and 91.8% of the thyroid lesions with diffuse microcalcifications developed central lymph node metastasis (27). Bai et al. (35) found that the presence of microcalcifications in PTC was significantly correlated with lymph node metastasis and clinical stage. Some cytokines, such as bone morphogenetic protein-1 and osteopontin, are highly expressed in tumors with microcalcifications (36), which are associated with the invasiveness of PTC. However, the results of this study showed that microcalcification was only statistically significant in univariate analysis, as multivariate analysis did not indicate that microcalcification was an independent risk factor for CLMN. The relationship between microcalcifications and CLNM needs to be further studied.

Hashimoto’s thyroiditis is a risk factor for the development of PTC (37). However, there is no consensus on the relationship between Hashimoto’s thyroiditis and CLNM in PTC patients. Hashimoto’s thyroiditis is a protective factor against CLNM, as the probability of developing CLNM is lower in PTC patients with Hashimoto’s thyroiditis (38). Zhou et al. (39) reported that TPO Ab <1 kU/L was an independent risk factor for central lymph node metastasis, but this was not confirmed in the study of Qu et al. (40). In our present study, there was no significant difference in the incidence of CLNM between PTC patients with Hashimoto’s thyroiditis and PTC patients without a Hashimoto’s thyroiditis background. TPOAb was not an independent risk factor for CLNM. The differences in the results of these studies may be related to the differences in the samples of different studies. The study of Zhou et al. only included unifocal PTC, and only central lymph node metastasis was analyzed. In this study, we included both unifocal PTC and multifocal PTC, and included both central lymph node metastasis and LLNM in the statistical analysis.

The present study has some limitations. First, the establishment of the nomogram model was based on a retrospective study of single-center samples, which may have a selection bias. Second, relatively few study subjects were included in this study, so the established nomogram model still requires prospective and big data studies to further verify its accuracy. Third, this study only included patients with PTC in China, and the applicability of the results to other pathological types of thyroid cancer (such as follicular thyroid carcinoma and medullary carcinoma) or populations of other races or countries needs to be further explored.

In summary, male sex, age <45 years, multifocality, maximum tumor diameter > 20 mm, extracapsular invasion, ambiguous boundary, and US suggested CLNM were independent risk factors for CLNM in patients with PTC. This study successfully established a nomogram model for predicting CLNM of PTC, which can help in the preoperative quantitative prediction of CLNM and should be considered in the determination of surgical procedures.

## Data Availability Statement

The original contributions presented in the study are included in the article/supplementary material. Further inquiries can be directed to the corresponding author.

## Ethics Statement

The studies involving human participants were reviewed and approved by the Ethics Committee of the People’s Hospital of Guangxi Zhuang Autonomous Region. Written informed consent to participate in this study was provided by the participants’ legal guardian/next of kin.

## Author Contributions

All authors listed have made a substantial, direct, and intellectual contribution to the work, and approved it for publication.

## Funding

This study was supported by the National Natural Science Foundation of China (No. 81660292, 81260223), Guangxi Natural Science Foundation project (2020GXNSFAA259014), and Guangxi medical high-level personnel training “139” project. The funders had no role in study design, data collection and analysis, decision to publish, or preparation of the manuscript.

## Conflict of Interest

The authors declare that the research was conducted in the absence of any commercial or financial relationships that could be construed as a potential conflict of interest.

## Publisher’s Note

All claims expressed in this article are solely those of the authors and do not necessarily represent those of their affiliated organizations, or those of the publisher, the editors and the reviewers. Any product that may be evaluated in this article, or claim that may be made by its manufacturer, is not guaranteed or endorsed by the publisher.
